# Polymeric Nanoparticles of Brazilian Red Propolis Extract: Preparation, Characterization, Antioxidant and Leishmanicidal Activity

**DOI:** 10.1186/s11671-016-1517-3

**Published:** 2016-06-17

**Authors:** Ticiano Gomes do Nascimento, Priscilla Fonseca da Silva, Lais Farias Azevedo, Louisianny Guerra da Rocha, Isabel Cristina Celerino de Moraes Porto, Túlio Flávio Accioly Lima e Moura, Irinaldo Diniz Basílio-Júnior, Luciano Aparecido Meireles Grillo, Camila Braga Dornelas, Eduardo Jorge da Silva Fonseca, Eduardo de Jesus Oliveira, Alex Tong Zhang, David G. Watson

**Affiliations:** Quality Control Laboratory of Drugs and Medicines, Postgraduate Program in Pharmaceutical Sciences, School of Nursing and Pharmacy, Federal University of Alagoas, Alagoas, Avenida Lourival Melo Mota, s/n Campus A. C. Simões, University City, Tabuleiro dos Martins, 57072-900 Maceió, Alagoas Brazil; Quality Control of Drugs Laboratory (LCQMed), Postgraduate Program of Pharmaceutical Science, Department of Pharmacy, Federal University of Rio Grande do Norte, Rua General Cordeiro de Farias S/N, Petrópolis, Natal, RN 59010-180 Brazil; Laboratory of Pharmaceutical analysis, Postgraduate Program of Pharmaceutical Science, Pharmacy College, Federal University of Vales do Jequitinhonha e Mucuri, Campus JK, Rodovia MGT 367 Km 583, n° 5000, Alto da Jacuba, Diamantina, Minas Gerais 39100-000 Brazil; Department of Pharmaceutical Science, Strathclyde Institute of Pharmacy and Biomedical Sciences, University of Strathclyde, 27 Taylor Street, Glasgow, G4 0NR UK

**Keywords:** Red propolis extract, PCL-pluronic nanoparticles, SEM analysis, Thermal analysis, ATR-FTIR, UPLC-DAD, Antioxidant activity, Leishmanicidal activity

## Abstract

The ever-increasing demand for natural products and biotechnology derived from bees and ultra-modernization of various analytical devices has facilitated the rational and planned development of biotechnology products with a focus on human health to treat chronic and neglected diseases. The aim of the present study was to prepare and characterize polymeric nanoparticles loaded with Brazilian red propolis extract and evaluate the cytotoxic activity of “multiple-constituent extract in co-delivery system” for antileishmanial therapies. The polymeric nanoparticles loaded with red propolis extract were prepared with a combination of poly-ε-caprolactone and pluronic using nanoprecipitation method and characterized by different analytical techniques, antioxidant and leishmanicidal assay. The red propolis nanoparticles in aqueous medium presented particle size (200–280 nm) in nanometric scale and zeta analysis (−20 to −26 mV) revealed stability of the nanoparticles without aggregation phenomenon during 1 month. After freeze-drying method using cryoprotectant (sodium starch glycolate), it was possible to observe particles with smooth and spherical shape and apparent size of 200 to 400 nm. Attenuated total reflectance Fourier transform infrared spectroscopy (ATR-FTIR) and thermal analysis revealed the encapsulation of the flavonoids from the red propolis extract into the polymeric matrix. Ultra performance liquid chromatography coupled with diode array detector (UPLC-DAD) identified the flavonoids liquiritigenin, pinobanksin, isoliquiritigenin, formononetin and biochanin A in ethanolic extract of propolis (EEP) and nanoparticles of red propolis extract (NRPE). The efficiency of encapsulation was determinate, and median values (75.0 %) were calculated using UPLC-DAD. 2,2-Diphenyl-1-picryhydrazyl method showed antioxidant activity to EEP and red propolis nanoparticles. Compared to negative control, EEP and NRPE exhibited leishmanicidal activity with an IC50 value of ≅38.0 μg/mL and 31.3 μg/mL, 47.2 μg/mL, 154.2μg/mL and 193.2 μg/mL for NRPE A1, NRPE A2, NRPE A3 and NRPE A4, respectively. Nanoparticles loaded with red propolis extract in co-delivery system and EEP presented cytotoxic activity on *Leishmania (V.) braziliensis*. Red propolis extract loaded in nanoparticles has shown to be potential candidates as intermediate products for preparation of various pharmaceutical dosage forms containing red propolis extract in the therapy against negligible diseases such as leishmaniasis.

**Electronic supplementary material:**

The online version of this article (doi:10.1186/s11671-016-1517-3) contains supplementary material, which is available to authorized users.

**Graphical Abstract:** Some biochemical mechanisms of cellular debridement of *Leishmania (V.) braziliensis* species by the flavonoids of red propolis extract (EEP) or NRPE loaded with red propolis extract

## Background

Propolis is a biotechnological product with biological activities produced by bees of the species *Apis mellifera* from plant exudates which are collected and added to organics or salivary secretions. Propolis has been widely used in alternative and traditional medicine to treat several diseases. The presence of secondary metabolites from plants like phenolic acids, phenolic esters, flavonoids, clerodanes, lupeones, propolones, prenylated benzophenones have been identified in different propolis around the world and are responsible for the biological activities in propolis raw material [1]. These compounds in whole are considered a “multiple-constituent extract” from a natural product and has a multi-target purpose in biological systems [2].

Propolis have presented diverse pharmacological activities amongst them antioxidant [3], antimicrobial [4, 5], antifungal [6, 7], antiviral [3], antiparasite [8, 9], anti-inflammatory [3, 4], anticancer [1, 10–16] and wound healing [17, 18], but its biological activity depends on its particular chemical composition and some favourable conditions such as bee species and its genetic variability, geographic area, biodiversity of plant species around hives, climate and sazonality and presence of water around the hives specially in semiarid area.

Recent studies that sought to characterize Brazilian red propolis found molecules, such as elemicin, isoelemicin, methyl isoeugenol, methyl eugenol, formononetin, biochanin A, isoliquiritigenin, liquiritigenin, medicarpin, homopterocarpan, quercetin and vestitol, that allow it to be distinguished from other types of Brazilian propolis [3, 19, 20]. The red propolis present the unique characteristics of chemical composition and multiple biological activities in addition to standardized production in the areas of mangroves swamps of Alagoas, Brazil, respecting a environment conservation policy of the Atlantic rainforest. Then, the production of red propolis raw material and hydroalcoholic extract is part of a production chain of apiceuticals and bioproducts that promotes sustainable development in the region of mangroves and lagoons being awarded the geographical indication label with the designation of origin.

Nanoparticles have been studied since early 1990s and used as drug nanocarriers and are be able to be encapsulated inside nanocarriers or onto the surface of small nanocarrier molecules, genes, biopharmaceuticals and diagnostic and imaging agents [21–23]. The nanocarriers commonly present different matrix systems (nanostructured materials) and an architecture composed of polymers (polymeric nanoparticles), liposomes (solid lipid nanoparticles, cubosomes, niosomes, spherulites etc.) [21, 22, 24], metal nanoparticles (silicon nanoparticles, gold nanoparticles, silver nanoparticles, magnetic nanoparticles), carbon nanotubes and quantum dots (diagnostic image agents) [23, 25–27].

But polymeric nanoparticles and liposomes are amongst the most accepted delivery vehicles of drugs with clinical applications in pharmaceutical area and approved as generally recognized as safe (GRAS). Polymeric nanoparticles reached this status due to biocompatibility, non-toxicity to biological systems, biodegradability, stability during storage, controlled release, target delivery, resulting in higher therapeutic efficacy [21, 25, 28, 29].

Nanoparticles can be prepared by different techniques including solvent emulsification-evaporation, high pressure homogenization, high-speed stirring, ultrasonication, microemulsion using spray-drying, nanoprecipitation and others [29–31]. Preparation and characterization of nanoparticles loaded with isolated drugs [32], vaccines [33] or isolated phytomedicine (kampferol, luteolin, cathechin, quercetin) [34, 35] and propolis extract [36] are applied in cancer therapy [37–39] and other diseases [40] including a negligible disease such as leishmaniasis [41, 42]. However, there are few papers describing herbal extract encapsulated in nanoparticles [43–49]. Few studies describe the use of propolis extracts with effective leishmanicidal effect [50]. There is also a current trend in the use of conventional therapy using the drug of first choice in association [51] or drug of first choice combined with phenolic compounds for treatment of various diseases resulting in enhancement of therapeutic efficacy or reducing the side effect of drugs used in the therapy of diseases such as leishmaniasis [52].

There is a great diversity in a nanoparticulate system to transport for target tissues the drug-drug or drug combined with phytochemicals [53, 54]. The development of “multi-compound extract in co-delivery system” for target tissues arises due to the high prevalence of the number of cases of cancers [55] and the increase in drug-resistance during leishmaniasis treatment worldwide [56–59].

The use of bee products and propolis comes from ancient times to present days, and there is an increasing interest to standardize apiceutical products for use in the pharmaceutical and cosmetic industries. It is necessary to carry out complex and diverse scientific studies of dose, composition, formulation stability and therapeutic activity of this complex mixture of substances of different phytochemical classes to ensure the efficacy, safety, quality and productivity. Currently, pharmaceutical industries have extensively explored the use of nanoparticles for “drugs in co-delivery system” in therapy against various diseases especially for the treatment of cancer as well as cardiovascular diseases, metabolic syndrome diseases and also negligible diseases. Thus, this study aimed to explore the potential of the propolis extract loaded in polymeric nanoparticles containing a pool of flavonoids and phenolic compounds which may act in a synergistic action for treating chronic and neglected diseases previously such as leishmaniasis.

The aim of the present study was to prepare and characterize polymeric nanoparticles loaded with Brazilian red propolis extract and access the antioxidant and leishmanicidal activity of “multiple-constituent extract in co-delivery system” present in red propolis extract loaded in a nanoparticulate system.

## Methods

### Propolis Sample

Red propolis raw material (150 g) was collected from Marechal Deodoro city, state of Alagoas, Brazil. Propolis was collected from the Ilha do Porto apiary with the following geographical coordinates: south latitude 9° 44.555′, west latitude 35° 52.080′ and height of 18.1 m above sea level. The access and transportation of Brazilian red propolis was previously authorised by regulatory agencies controlling Brazilian Genetic Heritage and Biodiversity Conservation with protocol number of acceptance 010124/2012-8.

### Reagents

Poly-ε-caprolactone (P.M 10.000), pluronic F-108 copolymer (poly(ethylene glycol)-block-poly(propylene glycol)-block-poly(ethylene glycol) (P.M. 14.000), and 2,2-diphenyl-1-picryhydrazyl (DPPH) reagents were purchased from Sigma-Aldrich (St. Louis, MO, USA). Analytical standards: pinobanksin, isoliquiritigenin, formononetin and biochanin A were purcharsed from Sigma-Aldrich (St. Louis, MO, USA), and liquiritigenin was acquired from Extrasynthese (Lyon, France). HPLC-grade acetonitrile was purchased from J. T. Baker Mallinckrodt-Avantor (NJ, USA). Schneider for insect medium was purchased from Sigma-Aldrich (St. Louis, MO, USA). Biphasic medium Schneider’s/Novy-McNeal-Nicolle, supplemented with 10 % of foetal bovine serum (FBS), 50 U of penicillin/mL and 50 μg of streptomycin/mL was purchased from Sigma-Aldrich (St. Louis, MO, USA).

### Preparation of Ethanolic Extract of Propolis (EEP)

Raw propolis (250 g) was manually grounded and placed in a flask with 600 mL of 80 % ethanol, which was placed on an agitator (Thornton, Model T14, USA) for 48 h. Then, the macerate (the liquid portion) was removed using a pipette, and the solid portion (wax) was discarded. The macerate was mixed with 600 mL of 80 % ethanol in a glass flask and placed on the agitator for 24 h. Then, the resulting macerate was mixed again with 600 mL of 80 % ethanol and left for 24 h without agitation.

Next, the macerate was removed using a pipette, filtered through filter paper and subjected to distillation under reduced pressure in a rotary evaporator (Fisatom, São Paulo, Brazil) in a water bath at temperature 40–50 °C, pressure 650 mmHg and speed 80 rpm to remove the solvent. The EEP was then placed in a glass container and left for approximately 3 days for the residual solvent to evaporate; as a result, a solid mass (162 g) with viscous appearance was obtained.

### Preparation of Nanoparticles Loaded with Nanoparticles of Red Propolis Extract (NRPE)

Five nanoparticles compositions containing red propolis extract were prepared using the polycaprolactone (PCL)-pluronic polymeric matrix (NRPE A1, NRPE A2, NRPE A3, NRPE A4, NRPE A5). Placebo composition (nanoparticles without red propolis extract) was also prepared. Table 1 summarizes the components present in these nanoparticles compositions.

**Table 1:** Compositions of nanoparticles loaded with red propolis extract (NRPE) using a nanocarrier in matrices system PCL-pluronic

	Composition (%)					
Components	NRPE A1	NRPE A2	NRPE A3	NRPE A4	NRPE A5	Placebo
Organic phase						
PCL	54.0	47.0	27.0	57.0	60.0	54.0
Red propolis extract	20.0	30.0	60.0	14.4	10.0	-
Aqueous phase						
Pluronic	26.0	23.0	13.0	28.6	30.0	46.0

The colloidal suspensions of nanoparticles were obtained by the preformed polymer interfacial deposition method also called nanoprecipitation preformed polymer [60]. The components of the organic and aqueous phases were weighed, placed in individual beakers and subjected to sonication until complete dissolution (15 min). Then, the organic phase (500 μL) was poured into the aqueous phase (50 mL) and vortexed for 1 min. The suspensions of polymeric nanoparticles containing red propolis extract (NRPE) were submitted to the characterization assays.

The NRPE were centrifuged for 15 min under a rotation speed of 5000 rpm in a small centrifuge for Eppendorf tubes (MiniSpin model) to form a solid pellet of non-rigid consistency in the bottom of the Eppendorf tube, which was later transferred to a 10-mL vial. The supernatant, containing acetone and water, was discarded, and nanoparticles were diluted with milli-Q water (1:5, *v*/*v*) and subjected to freeze-drying using three methods of drying. The suspension of nanoparticles were dried at room temperature (method A), by slow freeze method in freeze at −20 °C (method B) and by fast freeze method using liquid nitrogen (method C) to form the solid nanoparticles. The same procedure was performed for placebo composition (nanoparticles without red propolis extract, NRPE placebo).

### Freeze-Drying of Nanoparticles Loaded with Red Propolis Extract

The suspensions of red propolis nanoparticles were submitted to two freezing-drying processes: (1) slow freezing, in which the suspensions of red propolis nanoparticles were placed in a freezer at −20 °C for a period between 48 and 120 h and immediately transferred to freeze-dryer to perform the drying process for a period between 24 and 72 h (method B); and (2) fast freezing with liquid nitrogen at −196 °C for a period between 10 and 20 min and immediately transferred to a freeze-dryer to perform the drying process for a period between 24 and 36 h (method C).

Cryprotectant agents such as colloidal silicon dioxide from 0.1 to 10 %, sucrose between 1 and 15 %, glucose between 1 and 15 %, a solution containing combination of colloidal silicon dioxide and sodium starch glycolate (1:1, *w*/*w*) in concentration ranges between 10 and 30 % and sodium starch glycolate solution from 0.10 to 30 % were used.

The freeze-dryer used to dry the red propolis nanoparticles was a Terroni equipment®, LD 1500 model (São Paulo, Brazil), which comprises three shelves in a drying chamber, a condenser to −43 ± 5 °C and a vacuum pump. The equipment used for freeze-drying showed temperature stability and low pressure in the condenser. The system pressure remained below 300 μHg when the suspensions of nanoparticles are presented in solid state.

The EEP and nanoparticles loaded with red propolis extract were subjected to physicochemical characterization, investigation of antioxidant activity and leishmanicidal assay using *Leishmania (V.) braziliensis* species.

### Characterization of Suspension of Nanoparticles Loaded with Red Propolis Extract

#### Particle size, Zeta Potencial and pH

The determination of average diameter and polydispersion index of nanoparticles in suspension were made using dynamic light scattering. The suspensions were fourfold diluted in Milli-Q water and analysed in Zetasizer apparatus, model Nano-ZS from Malvern. The results were determined by the average of two cycles of 20 scans. The zeta potential of the nanoparticles suspension were obtained by the eletrophoretic mobility technique in the zetasizer apparatus in which the samples were diluted fourfold with Milli-Q water and results were expressed in millivolts (mV) from the average of two cycles of 20 scans. The nanoparticles pH determinations were performed using a previously calibrated glass electrode, and the measurements were carried out directly from nanoparticles suspension without any dilution.

### Solid State Characterization of Red Propolis Extract Loaded in Nanoparticles

#### Thermal Analysis

The calorimetric curves of polymeric nanoparticles containing red propolis extract were obtained in a differential scanning calorimeter, model DSC 50 from Shimadzu (Tokyo, Japan), using a mass of (5.0 ± 10 % mg), which were packed in aluminium capsules hermetically sealed. The heating rate was 10 °C min^−1^ in the temperature range 30–400 °C under an atmosphere of nitrogen and flow rate of 50 mL min^−1^. The DSC was calibrated using indium and zinc standards according to the Shimadzu suggested procedures.

#### ATR-FTIR

The nanopolymeric system loaded with propolis extract in solid state was subjected to attenuated total reflectance (ATR)-FTIR analysis, which was performed by ATR in the wavenumber range 4000 to 400 cm^−1^ and 64 scans. The results were expressed in infrared transmittance percentage. The equipment used was a Thermo Scientific coupled to the Ommic software for data acquisition. EEP and NRPE including placebo were analysed.

#### SEM Analysis

Scanning electron microscopy images were analysed to confirm the morphology and approximate size of nanoparticles in solid state. The compositions of red propolis nanoparticles were fixed on stubs with double carbon tape and covered by a gold film during the metallization process with 10 mA for 7 min in a System Sanyu Electron, Quick Coater Model SC-701. SEM micrographics have been taken from Shimadzu microscope (SSX-550 Superscan model).

### Determination of markers in Polymeric Nanoparticles Using UPLC-DAD

The identification and quantification of flavonoids in EEP and NRPE (in solid state) were performed using a ultra performance liquid chromatography coupled with diode array detector (UPLC-DAD) from Shimadzu consisting of the following modules: a high-pressure pump (Model LC-20ADXR), degasser (model DGU-20A3R), Autoinjector (model SIL-20AXR), oven chromatographic column, photodiode array detectors (model EPDM-20A) and fluorescence detector (RF-20A model), a controller (model CBM-20A) and a Shimadzu Labsolution software. The separation of flavonoids occurred using a reversed-phase column (C_18_, 150 × 4.6 mm; 5 μm), a mobile phase that consisted of solvent A (Milli-Q water) and solvent B (acetonitrile), pumped at a flow rate of 0.3 mL/min. The initial elution gradient consisted of water (70 %) and acetonitrile (30 %) with a variation of the percentage of acetonitrile to 100 % in 40 min followed by an isocratic condition with acetonitrile (100 %) up to 53 min and return to the initial condition at 54 min, followed by isocratic conditions acetonitrile (30 %) up to 60 min. This long method was developed in order to wash the column during the analysis with 100 % of acetonitrile and avoid lack of accuracy and precision during the efficiency of entrapment assay and to avoid column fouling and excessive pressure buildup by irreversible retention of non-polar compounds (terpenes and guttiferones present in red propolis extract).

Analytical standards were exactly weighed (2.0 mg) transferred to 10-mL volumetric flasks to obtain a concentration of 200 μg/mL. The working solutions were diluted to obtain the final calibration curve concentrations of 7.50, 5.00, 2.50, 1.00, 0.50 and 0.15 μg/mL. These calibration curves were used for determining the content (assay) of flavonoids in the extract (EEP) and NRPE loaded with red propolis extract. The identification of flavonoids was performed by retention time comparison with analytical standards using the same analytical conditions on the same working day.

### Efficiency of Encapsulation (%)

The efficiency of encapsulation was also performed using these same analytical conditions and with five markers (liquiritigenin, pinobanksin, formononetin, isoliquiritigenin, biochanin A) present in the EPP, which were determined in NRPE following the same separation conditions. The propolis extract (100 mg) as a solid (<1 % solvent) was solubilized in absolute ethanol with ultrasonic bath (5 min) and transferred to a volumetric flask (10 mL) to obtain a concentration of 10 mg/mL. A further dilution step was performed to obtain working solution of 1 mg/mL, and then final dilutions at 250 μg/mL were performed. These solutions were filtered through filter units 0.22 μm, and (2 μL) were injected in the UPLC-DAD system.

The efficiency of encapsulation (%) of flavonoids loaded in NRPE was performed by solubilizing the solid nanoparticles in a system of solvents consisting of acetone/ethanol (6:4, *v/v*) and also in the system of solvent water/ethanol (7:3, *v*/*v*) to verify flavonoid concentration outside non-encapsulated nanospheres (%). Nanoparticles loaded with red propolis extract (including NRPE placebo) were weighed and solubilized to obtain a concentration of 1.0 mg/mL. They were then diluted to a concentration corresponding to 250 μg/mL. The red propolis extract was also analysed in the same concentrations, and the efficiency of encapsulation (%) of each flavonoid in red propolis extract was determined using the following equation:

$$ \mathrm{Efficiency}\ \mathrm{of}\ \mathrm{encapsulation}\ \left(\%\right) = \left(\frac{\mathrm{Concentration}\ \mathrm{of}\ \mathrm{flavonoid}\ \mathrm{in}\ \mathrm{NREP}}{\mathrm{Concentration}\ \mathrm{of}\ \mathrm{flavonoid}\ \mathrm{in}\ \mathrm{E}\mathrm{E}\mathrm{P}}\right) \times 100 $$

### Antioxidant Activity of the Polymeric Nanoparticles Using DPPH Method

Quantitative assessment of the antioxidant activity of EEP and NRPE were performed according to the methods described in the literature [61] with few modifications. The inhibition of free radical DPPH by the samples was monitored by measuring the decrease in absorbance of solutions with different concentrations. The solvent ethanol was used as blank. A solution of DPPH (3 mM) was prepared transferring 0.0118 g of DPPH reagent to a 100-mL volumetric flask with ethanol.

The EEP and NRPEs were prepared at an initial concentration of 1.0 mg/mL in the solvent system acetone/ethanol (6:4, *v*/*v*). An aliquot of 400 μL was transferred to a volumetric flask of 5 mL, and then 2.0 mL of DPPH solution (3 mM) was added and diluted with ethanol until achieving final concentrations of 80.0 μg/mL. The reaction was left to develop in the dark at room temperature (25 °C) over 30 min. The absorbance readings were then performed with a spectrophotometer (Model UV-1240, Shimadzu, Kyoto, Japan) at 518 nm.

### In vitro Biological Assays

#### Antileishmanial Activity

##### *L. (V.) braziliensis* culture

The strains of *L. (V.) braziliensis* (IOC-L0566 - MHOM/BR/1975/M2903) from FioCruz (Recife, Brazil) [62, 41] promastigotes were cultured in vitro in biphasic medium Schneider’s/Novy-McNeal-Nicolle, supplemented with 10 % of FBS, 50 U of penicillin/mL and 50 μg of streptomycin/mL, incubated in a BOD camera at temperature of 26 °C.

##### Antileishmanial In vitro Assay

Sample preparation: previously, EEP and NRPE were exactly weighed and solubilized with DMSO/H_2_O (Milli-Q water) (75:25,*v*/*v*) to obtain a stock solution of 10,000 μg/mL. Using this solvent system, EEP was completely solubilized but NRPE was only partially solubilized. Several work solutions were obtained transferring aliquots of EEP or NRPE stock solution and diluting with Schneider’s medium supplemented with 10 % BFS to obtain volumes of 1 mL to each concentration at the range between 1000 and 5 μg/mL. In this way, the concentration of DMSO solvent did not exceed 0.5 %, and at this concentration, DMSO had no deleterious effect to the parasites [63].

Antileishmanial activity was carried out according to Rocha et al. [64]. Aliquots of (100 μL) of the cultured procyclic promastigotes cells of the parasite (at a concentration of 4 × 10^6^ cells/mL) immersed in the Schneider’s medium with 10 % BFS in 96-well microplates were incubated for a 24-h period in BOD camera at 26 °C with 100 μL of samples (EEP and NRPE) at the concentrations of 1000, 500, 400, 350, 300, 250, 200, 160, 100, 80, 60, 50, 40, 30, 20, 10 and 5 μg/mL. Afterwards, the morphology and parasites viability were evaluated in an inverted microscope (Olympus - IX70) to all concentrations tested, and then the parasite number was determined through optical microscope in the ×40 objective using a Neubauer chamber to express the antileishmanial effect. The results were expressed as the mean parasite growth inhibitory concentration (IC_50_).

### Statistical Analyses

All results are presented as mean ± standard deviation for, particle size, size distribution, zeta potential of NRPE, (%) efficiency of entrapment (*n* = 3) using UPLC-DAD and antioxidant activity by DPPH method. The mean ± standard deviation of the mean (SDM) relative to the percentage (normalized graphs) of cell growth inhibition and parasite growth inhibition were used to estimate the IC_50_ using non-linear regression with the software GraphPad Prism, version 5.0. The significance level was set as *p* < 0.05.

## Results and Discussion

### Characterization of Suspension of Nanoparticles Loaded with Red Propolis Extract

#### Particle Size, pH and Zeta Potential

The results for the assays performed for suspensions of nanoparticles (pH, particle size, polydispersity index and zeta potential) of NRPE are shown in Table 2 and confirmed the characteristics of a nanoparticulate system. The suspensions of NRPE presented with a homogeneous appearance macroscopically, opaque with Tyndall effect and light pink to intense pink coloured, depending on the concentration of the propolis extract loaded in nanoparticles. The opaque appearance and presence of the Tyndall effect in suspensions of nanoparticles can be indicative of the formation of nanospheres by nanoprecipitation method and can be explained by the Gibbs-Marangoni effect [65].

**Table 2:** Particle size and polydispersion index and zeta potential and pH of nanoparticles loaded with red propolis extract in suspension

Composition	Particle size (nm)	Polydispersion index (PDI)	Zeta potential (mV)	pH
NRPE placebo	279.6 ± 1.4	0.169	−33.5 ± 6.06	6.0 ± 0.1
NRPE A1	280.2 ± 8.7	0.089	−26.8 ± 4.64	6.1 ± 0.1
NRPE A2	262.2 ± 6.60	0.128	−18.6 ± 3.66	6.1 ± 0.2
NRPE A3	208.5 ± 4.79	0.115	−12.7 ± 5.24	5.8 ± 0.2
NRPE A4	225.2 ± 7.72	0.096	−24.2 ± 5.24	6.0 ± 0.1
NRPE A5	246.7 ± 9.2	0.113	−19.1 ± 4.61	6.1 ± 0.1

Mean values refer to the mean of three determinations ± standard deviation

Values of particle size in the nanometric scale varying between 208.5 and 280.2 nm with polydispersity index from 0.089 to 0.169 demonstrating unimodal model, i.e., monodisperse particles. The pH of the suspensions of nanoparticles (NRPE) was similar to the placebo with slightly acidic values between 5.83 and 6.15.The slightly acidic characteristic of suspension of nanoparticles can be attributed to the presence of the PCL polymer and the greater number of carboxyl end groups [66], and pH values between 4.6 and 6.0 were due to PCL polymer [67]. The pH is an important parameter to evaluate the stability of the nanoparticles in suspension and can be an indicator of polymer degradation or drug diffusion to aqueous medium.

The zeta potential varied from −18.6 to −26.8 mV, except NRPE A3 (−12.7 mV). Changes in the size of nanoparticles may be influenced by the nature and concentration of the polymer in the organic phase, polarity of solvent, the nature and concentration of the surfactants in the aqueous phase [68]. Chawla and Amiji [32] observed changes in particle size after removal of the pluronic F-68 surfactant with a significant increase in particle size. Therefore, the presence of pluronic in the nanoprecipitation process acts as a dispersant and also as a stabilizer. Solid materials have electric charge on its surface, after contact with a liquid. The high values of the zeta potential, either negative or positive (±30 mV), represent the achievement of a stable suspension, by repulsion between particles which prevents the occurrence of aggregation of the nanoparticles. The suspensions of nanoparticles were stable and dispersed, and no tendency to form aggregates was observed. In our studies, we monitored the stability of the suspensions of nanoparticles during 30 days and no aggregations or precipitation was observed, except for NRPE A3. Only NRPE A3 has developed precipitation after 10 days of storage.

Our strategies were prepare suspensions of nanoparticles or solid lipid nanoparticles loaded with red propolis extract and then incorporate it in cosmestic compositions (semisolid composition like cosmestic creams). Thus, these zeta potential values were sufficient to stabilize the nanoparticles of red propolis extract. Negative values for zeta potential are a desired condition to promote the permeation of molecules across the skin barriers especially for transdermal compositions. Nair et al. [69] reported that slightly negative or positive charges on the surface of the particles can control parameters such as the solubility and stability, as well as prevent loss of nanoparticles in undesired locations. In this way, it is a sine qua non condition for this natural product to maintain the multi-target purpose in biological systems. This stability of the suspensions of nanoparticles for our strategy (semisolid composition like cosmetic creams) is sufficient to stabilize the nanoparticles of red propolis extract.

### Characterization of Red Propolis Nanoparticles in Solid State

#### Thermal Analysis

**Fig. 1:**
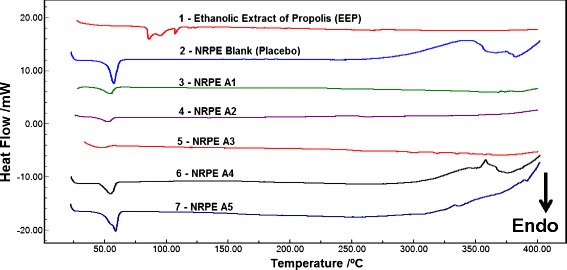
DSC curves of EEP and nanoparticles loaded with EEP in different compositions

The accompanying Fig. 1 shows DSC thermograms of the propolis extract, the polymeric coating matrix (PCL-pluronic) and NRPE compositions. The DSC thermogram of the red propolis extract showed four endotherm events at temperatures of 81.7, 92.0, 107.0 and 135.0 °C. Three endothermic peaks between 81.7 and 107.0 °C correspond to water volatilization, and the fourth endothermic peak at 135 °C refer to fusion processes of low molecular weight compounds like flavonoids in mixture and other phenolic compounds present in the propolis extract.

The polymers PCL and pluronic were submitted to DSC analysis and presented endothermic peak of fusion at 58 and 60 °C, respectively, while polymeric coating matrix showed an endothermic event at the melting temperature 60.5 °C and behaved similar NRPE to the NRPE compositions in the temperature range of 48 and 59 °C with no additional endothermic event at temperature ranges related to propolis extract (81.7, 92.0, 107 and 135 °C), suggesting an encapsulation phenomena of red propolis extract by the polymeric coating matrices.

#### ATR-FTIR

**Fig. 2:**
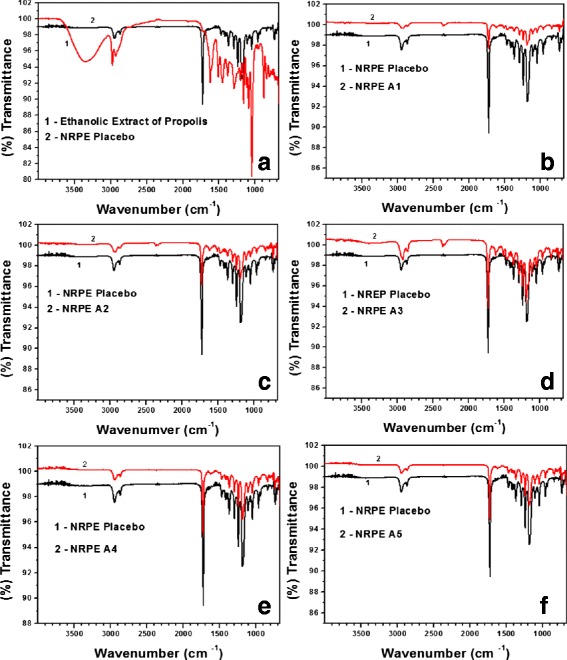
ATR-FTIR spectra of EEP and nanoparticles loaded with EEP in different compositions. Comparative ATR-FTIR spectra of NRPE Placebo and Ethanolic Extract of Propolis (**a**), NRPE Placebo and NRPE A1 (**b**), NRPE Placebo and NRPE A2 (**c**), NRPE Placebo and NRPE A3 (**d**), NRPE Placebo and NRPE A4 (**e**) and NRPE Placebo and NRPE A5 (**f**)

Figure 2 presents ATR-FTIR spectra of propolis extract, polymeric coating matrix and NRPE compositions. ATR-FTIR spectrum of the propolis extract showed typical hydrogen-bonded O–H stretch of phenolic compounds 3336 cm^−1^ (phenolic hydroxyl group), absorptions at 1617, 1496 and 1450 cm^−1^ corresponding to the C=C stretches of aromatic ring and the bands at 1045 cm^−1^ attributed to the stretch of aromatic ether C–O bond (for flavonoids) as well as the band at 877 cm^−1^ corresponding to the angular deformation outside the plane of aromatic C–H. The ATR-FTIR spectra of the polymeric coating matrices and NRPE compositions showed similar stretches at 2863 cm^−1^ and 1400–1340 cm^−1^ and are related to axial and angular stretches, respectively, for C–H of CH_2_. In addition, axial stretches of carbonyl aliphatic ketone (C=O) at 1725 cm^−1^ and axial and angular deformation C–(CO)–C at 1171 cm^−1^ were observed. Disappearance of the axial stretching of C=C (characteristic of aromatic ring) in flavonoids and phenolic compounds are present in red propolis extract. The spectroscopic infrared technique has proved encapsulation of red propolis extract during the processes of nanoprecipitation and drying using freeze-drying for NRPE.

### SEM Analysis

**Fig. 3:**
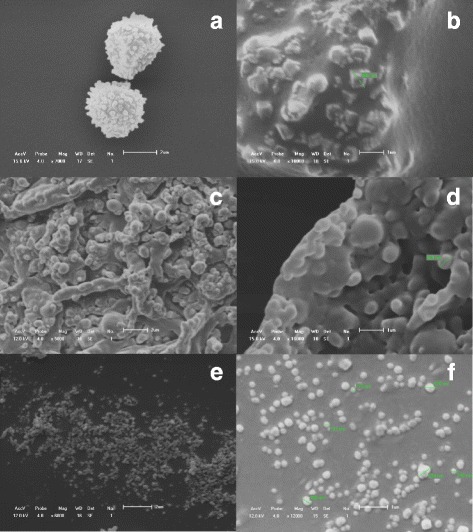
Scanning electron microscopy of NRPE in different freeze-drying conditions. **a**, **b** Method A of drying at room temperature in freeze-drying (NRPE A2). **c**, **d** Method B of drying in freeze-drying (NRPE A21). **e**, **f** Method C of drying in freeze-drying using cryoprotectants: colloidal silicon dioxide **e** and sodium starch glycolate **f** of the NRPE A4 composition

Figure 3 shows the scanning electron microscopy of NRPE A1, A2 and A4 using drying methods A and B. Photomicrographs with magnification of ×2000 (range 5 μm) and magnification up to ×10,000 (scale 1 μm) show submicron particles.

Figure 3a, b (NRPE A1 and NRPE A1) was obtained by the method of drying at room temperature (natural drying). Figure 3a, b shows photomicrographs of submicron particles (between 450 and 960 nm) obtained by drying process at room temperature (method A) with the formation of nanocrystals, and Fig. 3c, d shows scanning electron microscopy of NRPE A2 like submicron particles (between 400 and 960 nm) with the formation of amorphous aggregates when obtained using drying method B (slow freezing at freezer −20 °C).

Figure 3 shows scanning electron microscopy of NRPE A4 obtained by method C of drying. Micrographs with magnification of ×7000 (scale 2 μm) and magnification up to ×10,000 (scale 1 μm) showing the polymeric nanoparticles loaded with red propolis extract. Figure 3e, f (NRPE A4) was obtained by drying method C. Figure 3e shows a micrograph of the nanoparticles (200 and 350 nm) using colloidal silicon dioxide as cryoprotectant, and Fig. 3f shows a photomicrograph of the nanoparticles (between 175 and 320 nm) using a sodium starch glycolate as cryoprotectant. Method C presented spherical nanoparticles in nanometric scale and no aggregates.

### Determination of markers in Polymeric Nanoparticles Using UPLC-DAD

**Fig. 4:**
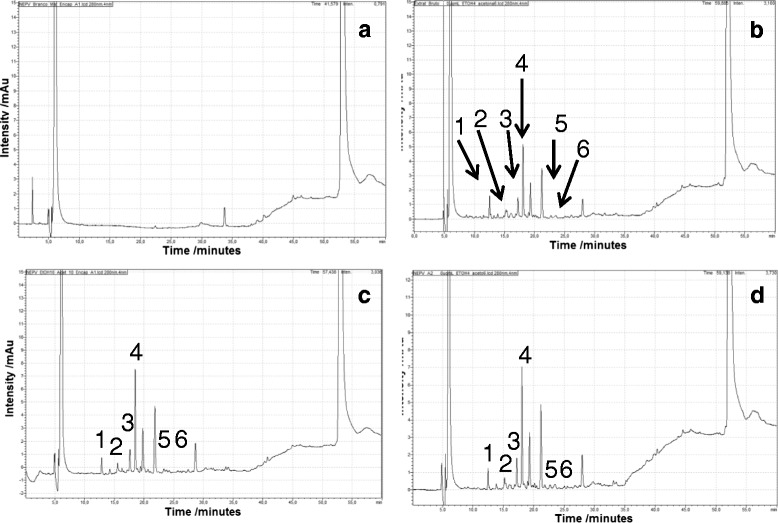
Determination of markers of red propolis extract in EEP and NRPE using UPLC-DAD. Chromatogram of the placebo (**a**), EEP (**b**), NRPE A1 (**c**) and NRPE A2 (**d**). Identification of flavonoids (*1*) liquiritigenin (*2*) pinobanksin, (*3*) isoliquiritigenin, (*4*) formononetin, (*5*) biochanin A and (*6*) pinocembrin at the wavelength of 280 nm

The chromatograms by UPLC-DAD in Fig. 4 show the polymeric coating matrices (placebo) (Fig. 4a), red propolis extract (Fig. 4b), NRPE A1 composition (Fig. 4c) and NRPE A2 composition (Fig. 4d). Identification of flavonoids and isoflavonoids present in the red propolis extract and NRPE compositions are shown. Chromatographic peaks for flavonoids in the polymeric coating matrice (placebo) were not observed (Fig. 4a). Figure 4b–d shows the presence of flavonoids (1) liquiritigenin (12.53 min), (2) pinobanksin (15.68 min), (3) isoliquiritigenin (17.26 min) (4) formononetin (18.13 min) (5) pinocembrin (23.12 min) and (6) biochanin A (23.81 min) at the corresponding retention times similar to those used as analytical standards during the development of chromatographic method. The method showed linearity, precision and accuracy in the concentration range between 0.15 and 5.0 μg/mL and was previously validated for six flavonoids identified as markers in red propolis extract and NRPE. It was possible to determine the concentration of five markers present in EEP and the nanoparticles loaded with propolis extract with intermediary precision of 1.61, 2.65, 3.00, 5.05 and 4.65 % for liquiritigenin, pinobanksin, isoliquiritigenin, formononetin and biochanin A, respectively (Additional file 1: Table S1). Accuracy of 8.28, 10.51, −7.43, −2.48 and 13.00 % was observed in the low limit of quantification (0.15 μg/mL), respectively, for the flavonoids cited in this order (Additional file 2: Table S2). Pinocembrin presented low levels in nanoparticles, and it was not used for determination of efficiency of entrapment assay. Table 3 shows the concentrations (μg/mL) obtained for each biomarker, and the data was used to determine the efficiency of entrapment and to establish the range of concentration of red propolis extract in biological assays. Variability on the results up to 13 % can be considered as a method variation and can be explained due to use of gradient method used for eluted flavonoids with different grade of polarity and the complexity of the sample (a pool of flavonoids).

**Table 3:** Determination of the markers present in EEP and nanoparticles loaded with red propolis extract using UPLC-DAD

	Concentration of flavonoids(μg/mL) ± SD^a^				
Composition	A	B	C	D	E
EEP	2.290 ± 0.760	0.330 ± 0.003	2.510 ± 0.010	2.960 ± 0.010	0.371 ± 0.010
NRPE A1	1.240 ± 0.040	0.329 ± 0.004	2.400 ± 0.040	1.740 ± 0.010	0.370 ± 0.004
NRPE A2	0.950 ± 0.010	0.238 ± 0.020	1.770 ± 0.020	1.740 ± 0.010	0.324 ± 0.003
NRPE A3	0.820 ± 0.01	0.200 ± 0.004	2.032 ± 0.001	1.860 ± 0.010	0.318 ± 0.001
NRPE A4	1.216 ± 0.004	0.240 ± 0.003	1.888 ± 0.001	2.032 ± 0.022	0.327 ± 0.002
NRPE A5	1.168 ± 0.003	0.230 ± 0.001	1.792 ± 0.031	2.002 ± 0.013	0.321 ± 0.002

*A* liquiritigenin, *B* pinobanksin, *C* isoliquiritigenin, *D* formononetin, *E* biochanin A

^a^Mean values refer to the mean of three determinations ± standard deviation

### Efficiency of Encapsulation (%)

Five marker compounds representing the major flavonoids found in the samples were used for determination of content and (%) efficiency of encapsulation. It was observed that liquiritigenin showed greater losses during the drying process of NRPE. The compositions NRPE A1, A4 and A5 showed higher content values and (%) efficiency of encapsulation, while NRPE A2 and A3 showed the greatest loss for liquiritigenin (Table 4). Greater percentage of encapsulation for the flavonoids isoliquiritigenin, formononetin and biochanin A was observed and was superior to 75 %, except in compositions NRPE A2 (Table 4). The percentage of non-encapsulated flavonoids was evaluated, and a high concentration of flavonoids on the surface of the nanoparticles for compositions NRPE A3 (41.48 %) and NRPE A2 (39.47 %) was observed (Table 5). Approximately the percentage of 40 % of flavonoids loaded in the nanoparticle can be retained on the outer walls of the nanoparticles, and this point is very important to maintain the biological activity of this apiceutical extract. This phenomenon occurred in all compositions but in the concentration of propolis extract was greater than 30 % (NRPE A2 and NRPE A3, both loaded with 40 and 50 % of red propolis extract, respectively) also due to minor proportion of the polymeric system (PCL-pluronic) required for encapsulating the flavonoids present in red propolis extract. A percentage of 70 % of the polymeric system (PCL-pluronic) and 30 % of red propolis extract was considered optimum to encapsulate flavonoids from red propolis and can be considered a limit to encapsulate the flavonoids of red propolis extract. A higher concentration of red propolis extract on the polymeric system provoked saturation of the system and reduced the efficiency of encapsulation. The NRPE A1, A4 and A5 demonstrated greater capacity of entrapment to the flavonoids of propolis extract compared with other compositions. The NRPE A2 and NRPE A3 displayed evidence of flavonoids adsorbed on the external surface of nanoparticles (Table 5).

**Table 4:** Efficiency of encapsulation (%) of flavonoid marker compounds present in the nanoparticles loaded with red propolis extract

	(%) Efficiency of encapsulation of flavonoids using UPLC-DAD^a^				
Composition	A	B	C	D	E
NRPE A1	53.0 ± 1.6	99.8 ± 1.7	95.6 ± 1.7	77.4 ± 1.7	99.8 ± 1.7
NRPE A2	41.3 ± 0.4	72.0 ± 0.9	70.5 ± 0.9	58.6 ± 0.5	82.1 ± 1.3
NRPE A3	38.0 ± 0.8	60.9 ± 1.3	103.6 ± 0.7	75.4 ± 0.6	85.7 ± 0.2
NRPE A4	57.3 ± 0.3	72.9 ± 0.8	96.6 ± 0.1	82.2 ± 1.4	88.3 ± 1.7
NRPE A5	54.9 ± 0.2	70.9 ± 0.2	91.5 ± 2.5	81.2 ± 0.8	86.4 ± 1.7

*A* liquiritigenin, *B* pinobanksin, *C* isoliquiritigenin, *D* formononetin, *E* biochanin A

^a^Mean values and standard deviation determinate in triplicate

**Table 5:** Efficiency of encapsulation (%) and non-encapsulation proportion (%) of the flavonoid marker compounds present in nanoparticles of red propolis extract

	(%) Efficiency of encapsulation of flavonoids using UPLC-DAD		
Composition	(%) E.E.	(%) N.E.	∑ (%E.E. + %N.E.)
NRPE A1	85.15	14.79ª	96.25
NRPE A2	65.74	39.47^b^	105.20
NRPE A3	72.76	41.48ª	114.24
NRPE A4	79.49	26.09^c^	105.58
NRPE A5	77.01	18.82^d^	95.83

Mean values and standard deviation determinate in triplicate

*E.E.* efficiency of encapsulation, *N.E.* non-encapsulated

^a^External determination of formononetin

^b^External determination of liquiritigenin + pinobanksin + isoliquiritigenin + formononetin

^c^External determination of liquiritigenin and formononetin

^d^External determination of liquiritigenin + isoliquiritigenin + formononetin

**Fig. 5:**
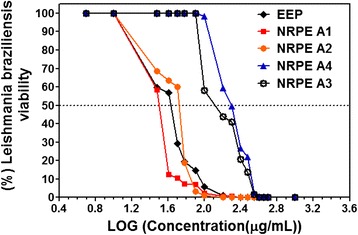
Determination of IC_50_ for leishmanicidal assay against *Leishmania (V.) braziliensis* using normalized graph

The compositions NRPE A1 and NRPE A2 showed a polymeric system PCL-pluronic that do not compromise the stability, the potency, or biological activity of flavonoids in propolis extract which are present inside or on the outer walls of the nanoparticles. No strong chemical interaction was shown; only physical interactions between embedded extract to the polymeric system were observed. The results of antioxidant activity and leishmanicidal activity demonstrate this statement (Table 5 and Fig. 5). The high content of PCL-pluronic system in relation to propolis extract affected the potency of the compositions NRPE A4 at the leishmanial assay (Table 5 and Fig. 5). The composition NRPE A3 demonstrated vulnerability to oxidative degradation by air or light during the drying process by freeze drying, evidenced by the darkening of the material thus obtained.

Generally, an apiceutical extract is a “multiple-component agent” and presents different chemical classes of compounds like phenolic acids, esters, flavonoids and terpenes, and each chemical class display a particular chemical reactivity and is likely to react with light, air, water and other extrinsic factors promoting rapid degradation or inactivation of biological activity. The polymeric encapsulation of red propolis extract was a successful strategy to protect this apiceutical extract against degradation and inactivating itself; besides, this encapsulation process was able to store the red propolis extract during 9 months in solid-state form.

### Antioxidant Activity of the Polymeric Nanoparticles Using DPPH Method

The NRPE displayed good antioxidant activity with inhibition values between 76.22 and 81.40 % for compositions and only 12.28 to 13.75 % for placebo sample (Table 6). The development of polymeric nanoparticles loaded with propolis extract solves a technological problem in the field of cosmetic science and phytocosmetics since the presence of phenolics in high concentration in these preparations can result in chemical incompatibilities, fast darkening of these semisolid preparations by the autoxidation phenomena between phenolic compounds and lipids from cosmetic excipients. Therefore, the nanoencapsulation of the apiceutical extract into inert polymeric systems such as polycaprolactone and pluronic is necessary to coat the flavonoids and protect them from possible autooxidative reactions favoured by the other cosmetic constituents. These results demonstrate that the compositions NRPE will act as agents that inhibit oxidative processes and act as antioxidants in biological systems. Antioxidant activity in vitro using DPPH assay may be the first indicator of action of these polymeric nanoparticles on the skin and associated structures functioning as skin anti-ageing agent, and inhibiting muco-cutaneous growth of parasites.

**Table 6:** Antioxidant activity (%AOA) of EEP and its nanoparticles loaded with red propolis extract

	(% AOA)		
Composition	(%)E.E.^a^	(%)N.E.^a^	∑(%E.E. + %N.E.)
Trolox	99.50	^a^	99.50
EEP	98.00	^a^	98.00
NRPE A1	81.40 ± 2.75	30.73 ± 1.04	112.13
NRPE A2	76.63 ± 6.86	27.18 ± 0.95	103.81
NRPE A3	79.51 ± 2.13	36.75 ± 1.33	116.26
NRPE A4	77.35 ± 2.98	38.10 ± 0.36	115.45
NRPE A5	76.22 ± 5.74	18.27 ± 1.77	94.49

Free radical DPPH sequestering activity (%) of the ethanolic extract of Brazilian red propolis and its nanoparticles loaded with red propolis extract

^a^Mean values refer to the mean of three determinations ± standard deviation

### In vitro Biological Assays

#### Antileishmanial Activity

The EEP and NRPE presented antileishmanial activity proven by IC_50_ values. The EEP showed an IC_50_ of 37.9 μg/mL (CI 95 % 33.57–43.11 μg/mL), and NRPE samples showed an IC_50_ of 31.34 μg/mL (CI 95 % 27.24–35.71 μg/mL) (NRPE A1), 47.23 μg/mL (CI 95 % 42.41–53.23 μg/mL) (NRPE A2), 154.2 μg/mL (CI 95 % 140.60–169.20 μg/mL) (NRPE A3) and 193.2 μg/mL (CI 95 % 187.60–201.40 μg/mL) (NRPE A4) (Fig. 5A). In general, the compositions of EEP-loaded into nanoparticles comprising 30 and 40 % of EEP maintained antileishmanial activity similar to the EEP in its original form. Leishmanicidal activity of the prepared nanoparticles has not been found to be a concentration-dependent response but is related to efficiency of encapsulation and ratio between polymeric matrix and extract of red propolis present in the nanoparticles. The NRPE A4 composition demonstrated the lowest amount of EEP and had the lowest leishmanicidal activity of these compositions. In general, the polymeric matrix (PCL-pluronic) has shown to be a controlled release system very efficient for release of drugs, and in this particular composition (NREP A4), the polymeric system served as a delayed release system by reducing the release of flavonoids present in the propolis extract against *L. braziliensis*. The NRPE A3 composition presented highest loss for markers liquiritigenin and formononetin, and the highest amount of markers determined outside of nanoparticles was observed (Tables 4 and 5).

Propolis extracts from different part of the world have been investigated against leishmania parasites and has demonstrated relevant leishmanicidal activity. Duran et al. [70] showed antileishmanial activity for two types of Turkey propolis extracts (propolis Hatay and Bursa propolis) and demonstrated an IC_50_ of 250 and 500 μg/mL. Another relevant study by Duran et al. [71] revealed good results for Adana propolis against leishmania parasite in concentrations above 250 μg/mL. Another type of Turkish propolis extract (Kayseri propolis) studied by Ozbilge et al. [72] showed excellent leishmanicidal activity against *Leishmania tropica* with an IC_50_ of 32 μg/mL.

Comparative study of the green propolis extracts from Brazilian and Bulgarian propolis extract carried out by Machado et al. [73] showed leishmanicidal activity against four different species of *Leishmania* (*amazonensis*, *braziliensis*, *chagasi* and *major*). Brazilian green propolis extract showed IC_50_ close to 49 μg/mL against *L. braziliensis*, *L. chagasi* and *L. major* species, while the Bulgarian propolis extract showed leishmanicidal activity for *L. amazonensis*, *L. chagasi* and *L. major* species with IC_50_ between 2.8 and 41.3 μg/mL. Excellent leishmanicidal activity of Bulgarian propolis can be explained by the presence of various flavonoids present in its extract as pinobanksin esters, pinocembrine, chrysin, some phenolic acids and phenolic acid esters [73]. Brief description of the scientific literature shows that some flavonoid aglycones such as quercetin, luteolin and fisetin have high leishmanicidal activity with IC_50_ values in the range from 0.6 to 0.8μg/mL. Flavonoid glycosides such as rutin, quercitrin and isoquercitrin have also demonstrated leishmanicidal activity. Scientific research by Da Silva et al. [74] showed that the Brazilian green propolis displays leishmanicidal activity in concentrations ranging from 5 to 100 μg/mL in in vitro cytotoxicity studies at the times 24, 96 and 168 h. Miranda et al. [75] demonstrated efficacy of combined therapy of complex Ru-NO (NO donor) and Brazilian green propolis extract in the treatment of American cutaneous leishmaniasis (ATL).

Brazilian red propolis extract with high concentrations of prenylated benzophenones has been evaluated in macrophages infected with *L. amazonensis* and demonstrated that a concentration of 25 μg/mL is capable of increasing the reductive activity of MTT being active against these intracellular parasites present in macrophages [76]. In our studies, the hydroalcoholic propolis extract showed antileishmanial activity against *L. braziliensis* with IC_50_ values ≅38.0 μg/mL, and the same results were obtained with polymeric nanoparticles loaded with propolis extract with IC_50_ values between 31.3 and 47.2 μg/mL for NRPE A1 and NRPE A2, respectively.

We have developed a method to evaluate only biological activity of nanoparticles (NRPE) against the parasite *L. (V.) braziliensis* that is a biological quality control method for these nanoparticles, and it is considered as an intermediary product (bulk form) during the production of semisolids (cosmetic creams). In our experiments, we proved similar efficacy of NRPE (nanoparticle loaded with red propolis) to EEP (ethanolic extract of red propolis) for NRPE A1 and NRPE A2, and these nanoparticles will deserve further development efforts aimed at a final composition (cosmetic cream) against cutaneous leishmaniasis. Previous studies using emulsionated curcumin [77] and phospholipid nanoparticles of ursolic acid [78] obtained using preparation techniques such as emulsification-evaporation and amphiphilic self-assembly was shown to affect parameters such as absorption, half-life and bioavailability when compared to the effect of the isolated substances. Thus, in the final formulation excipients could be taylored to modulate and improve pharmacokinetic parameters of natural products [69].

Our chormatographic study determined the presence of some isoflavonoids (formononetin, biochanin A), dyhydroflavonol (pinobanksin), chalcone (isoliquiritigenin) and flavonone (liquiritigenin), but formononetin, isoliquiritigenin and liquiritigenin were considered the major flavonoids present in the EEP and also in NRPE. It was previously shown that the presence of isoliquiritigenin, a chalcone, and other flavonoids and isoflavonoids present in EEP and in the prepared nanoparticulate extract (NRPE) can inhibit the activity of *L. (V.) braziliensis* through different biochemical targets such as cytoplasmic membrane and the mitochondrial respiratory complex. The propolis extract present a multitude of constituents that can act on multiple biochemical targets affecting the balance of complex cellular networks in a way that is currently not fully understood such as the multi target efficacy model proposed by CSERMELY et al. [2].

Recent studies showed that flavonoid chalcones have high activity in inhibiting the growth of parasites including *Leishmania*. Torres-Santos et al. [79] demonstrated that naturally occurring chalcones (dihydromethoxylated chalcone) alter the biosynthesis of sterols in *L. amazonensis* and promote ergosterol accumulation and cholesterol decrease. This alteration resulted into increase the membrane fluidity and toxic effect to the parasite with IC_50_ of 5.5 μM. Previous studies have shown a similar mechanism of action for amphotericin B (leishmanicidal drug) [80].

**Fig. 6:**
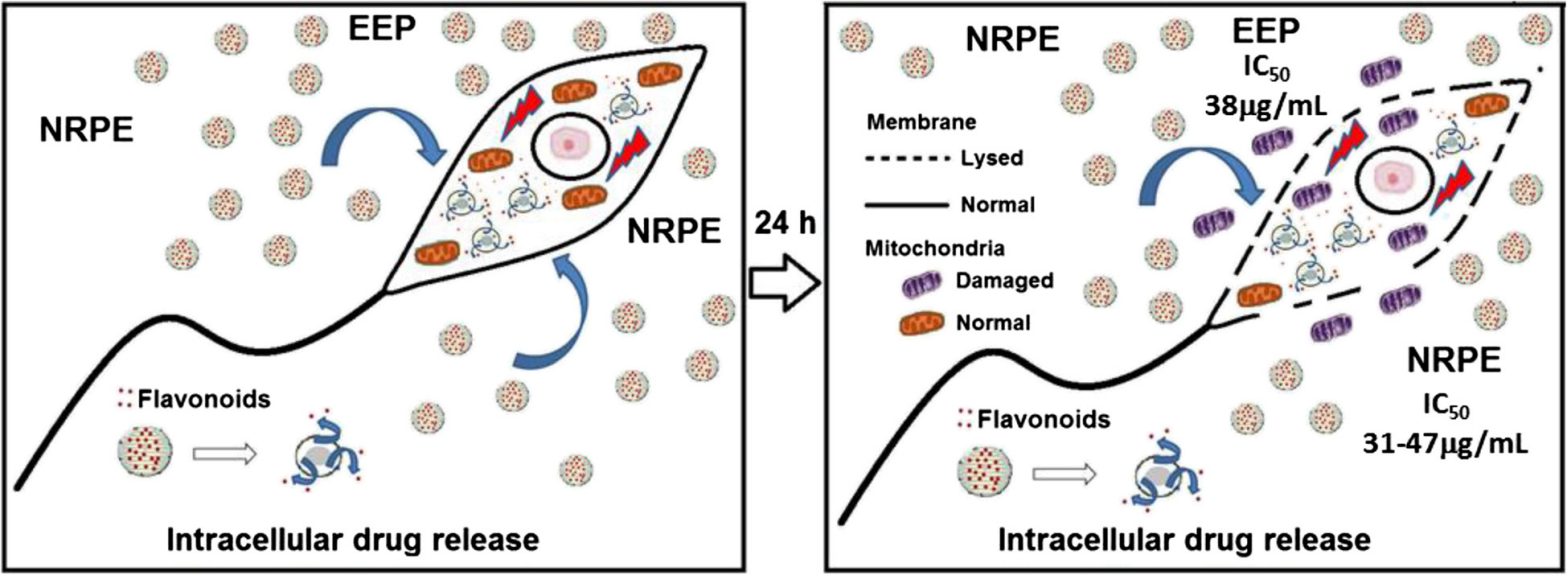
Some hypothesized biochemical mechanism of cellular debridement of *Leishmania (V.) braziliensis* species by the flavonoids of red propolis extract (EEP) or NRPE loaded with red propolis extract

Studies of Chen et al. [81] showed that chalcones (licochalcone A in specific study) can destroy the ultrastructure of the parasite’s mitochondria and inhibit mitochondrial respiration and activity of mitochondrial dehydrogenase enzymes, especially the activity of the parasite´s specific fumarate reductase (FDH). Some studies have presented a similar mechanism of action for the antileishmanial drug paromonycin, which binds to the mitochondrial 30S ribosomal subunit as well as the induction of respiratory dysfunction and depolarization of the mitochondrial membrane [82, 83]. Figure 6 summarizes these two hypothesized mechanisms of growth inhibition of leishmania using a pool of flavonoids in EEP or NRPE.

## Conclusions

Nanoparticles (200-800nm) loaded with red propolis extract were prepared and characterized both as suspensions and in solid state form, based on PLC-pluronic matrix system. This copolymeric matrix system was able to encapsulate different flavonoids from red propolis extract with particular characteristics of solubility and antioxidant activity.

Nanoparticles loaded with red propolis extract in multidrug co-delivery system and EEP presented cytotoxic activity on *L. braziliensis*. Red propolis extract loaded in nanoparticles was shown to be a potential candidate as intermediate products for preparation of various pharmaceutical dosage forms containing red propolis extract in the therapy against neglected diseases such as leishmaniasis.

## Abbreviations

%AOA, percentage of antioxidant activity; DMSO, dimethyl sulphoxide; EEP, ethanolic extract of propolis; NRPE, nanoparticles of red propolis extract; DPPH, 2,2-diphenyl-1-picryhydrazyl; UPLC-DAD, ultra performance liquid chromatography coupled with diode array detector.

## Additional files

Additional file 1: Table S1. Intra-day precision data for five flavonoids of red propolis extract. (DOCX 39 kb) Additional file 2: Table S2. Accuracy data for five flavonoids of red propolis extract. (DOC 32 kb)
