# Supplementary material for: Polymeric Nanoparticles of Brazilian Red Propolis Extract: Preparation, Characterization, Antioxidant and Leishmanicidal Activity
Source: Nanoscale Res Lett. 2016 Jun 17;11:301. doi: 10.1186/s11671-016-1517-3 (PMC4912519; doi:10.1186/s11671-016-1517-3)
Supplement: Additional file 1: Table S1. — Intra-day precision data for five flavonoids of red propolis extract. (DOCX 39 kb) [file 11671_2016_1517_MOESM1_ESM.docx]

**Type of article:** NanoExpress

Title Page

**Title: Title:** Polymeric nanoparticles of Brazilian Red Propolis Extract: Preparation, characterization, antioxidant and leishmanicidal activity

**Additional Tables**

**Additional Table 1**. Intra-day precision data for 5 flavonoids of Red Propolis Extract

| Concentration  μg/mL | Concentration(μg/mL)±RSD(%) | | | | |
| --- | --- | --- | --- | --- | --- |
|  | Liquiritigenin | Pinobanksin | Isoliquiritigenin | Formononetin | Biochanin A |
| 0.150 | 0.162±1.00 | 0.179±2.65 | 0.166±3.00 | 0.168±1.66 | 0.190±4.65 |
| 0.500 | 0.514±1.61 | 0.518±1.38 | 0.532±0.68 | 0.519±0.50 | 0.515±0.80 |
| 1.000 | 0.955±0.53 | 0.942±0.69 | 0.953±1.03 | 0.948±0.71 | 0.934±0.12 |
| 2.500 | 2.523±0.05 | 2.503±0.40 | 2.486±0.58 | 2.514±5.07 | 2.501±0.13 |
| 5.000 | 4.996±0.26 | 5.007±0.51 | 5.013±0.12 | 5.001±0.27 | 5.011±0.58 |
