# Supplementary material for: Polymeric Nanoparticles of Brazilian Red Propolis Extract: Preparation, Characterization, Antioxidant and Leishmanicidal Activity
Source: Nanoscale Res Lett. 2016 Jun 17;11:301. doi: 10.1186/s11671-016-1517-3 (PMC4912519; doi:10.1186/s11671-016-1517-3)
Supplement: Additional file 2: Table S2. — Accuracy data for five flavonoids of red propolis extract. (DOC 32 kb) [file 11671_2016_1517_MOESM2_ESM.doc]

**Additional Table 2**. Accuracy data for 5 flavonoids of Red Propolis Extract

| Concentration  μg/mL | Accuracy (%) | | | | |
| --- | --- | --- | --- | --- | --- |
|  | Liquiritigenin | Pinobanksin | Isoliquiritigenin | Formononetin | Biochanin A |
| 0.150 | 8.280 | 10.508 | -7.428 | 1.200 | 13.022 |
| 0.500 | 2.795 | 0.841 | 2.739 | -2.482 | -0.731 |
| 1.000 | -4.524 | -1.366 | 1.152 | -0.496 | -1.445 |
| 2.500 | 0.925 | -0.794 | -0.682 | 1.109 | -0.548 |
| 5.000 | -0.086 | 0.234 | 0.106 | -0.229 | 0.186 |
